# Efficacy and safety of risdiplam in adults with 5q-associated spinal muscular atrophy: a nationwide observational cohort study in Austria

**DOI:** 10.1016/j.eclinm.2025.103536

**Published:** 2025-09-26

**Authors:** Omar Keritam, Marcus Erdler, Bernhard Fasching, Gudrun Zulehner, Jakob Rath, Martin Krenn, Thomas Waldhör, Victoria Anna Gruber, Nadine Langweil, Christian Kiss, Theresa Antonia Griedl, Valeriu Gold, Julia Wanschitz, Anna Hotter, Vera E.A. Kleinveld, Corinne G.C. Horlings, Astrid Erber, Eva Schernhammer, Johannes Troger, Susanne Grinzinger, Petra Müller, Dieter Langenscheidt, Mika Rappold, Anna Wiesenhofer, Magdalena Gosk-Tomek, Florian Knipp, Simone Mahal, Günther Bernert, Matthias Baumann, Fritz Zimprich, Raffi Topakian, Christian Eggers, Stefan Quasthoff, Wolfgang Löscher, Hakan Cetin

**Affiliations:** a Department of Neurology, Medical University of Vienna, Vienna, Austria; b Comprehensive Center for Clinical Neurosciences and Mental Health, Medical University of Vienna, Vienna, Austria; c Department of Neurology, Klinik Donaustadt, Vienna, Austria; d Department of Epidemiology, Center for Public Health, Medical University of Vienna, Vienna, Austria; e Vienna General Hospital, Vienna, Austria; f Department of Neurology, Medical University of Graz, Graz, Austria; g Department of Neurology, Medical University of Innsbruck, Innsbruck, Austria; h Department of Epidemiology, Harvard T.H. Chan School of Public Health, Boston, MA, USA; i Department of Neurology, Klinikum Klagenfurt, Klagenfurt, Austria; j Department of Neurology, Paracelsus Medical University, Salzburg, Austria; k Department of Neurology, Academic Teaching Hospital Wels-Grieskirchen, Wels, Austria; l Department of Neurology, Landeskrankenhaus Rankweil, Rankweil, Austria; m Department of Paediatrics, Klinik Favoriten, Vienna, Austria; n Division of Paediatric Neurology, Department of Paediatrics I, Medical University Innsbruck, Innsbruck, Austria; o Department of Neurology, Johannes Kepler University, Linz, Austria

**Keywords:** Spinal muscular atrophy, SMA, Risdiplam, Observational study

## Abstract

**Background:**

Spinal muscular atrophy (SMA) is a genetic motor neuron disease marked by the progressive decline of motor function. Risdiplam, an orally administered *SMN2* splicing modifier, was approved for the treatment of 5q-associated SMA (5q-SMA) across all age groups. However, clinical trial data have primarily focused on paediatric populations, with limited evidence available for adult patients. This study aimed to evaluate the efficacy and safety of risdiplam in treatment-naïve adults with 5q-SMA in a real-world, multicentre setting.

**Methods:**

We conducted a nationwide, observational cohort study across eight neuromuscular centres in Austria. Patients aged ≥16 years at treatment initiation with genetically confirmed 5q-SMA, who were previously untreated and initiated risdiplam between December 2020 and September 2024 were eligible for inclusion if they had received risdiplam for ≥3 months and had functional motor assessments available at baseline (T0) and at least one follow-up. Functional outcomes were assessed at four predefined intervals after baseline: 3–<6 months (T1), 6–<12 months (T2), 12–<18 months (T3), and ≥18 months (T4). The primary outcome was the change from baseline in the Hammersmith Functional Motor Scale Expanded (HFMSE). Secondary outcomes included changes in the Revised Upper Limb Module (RULM), Amyotrophic Lateral Sclerosis Functional Rating Scale-Revised (ALSFRS-R), and 6-min walk test (6MWT). Adverse events were extracted from medical records.

**Findings:**

A total of 87 patients had received risdiplam, of whom 57 fulfilled the inclusion criteria and were included in this study. The median age at treatment initiation was 35.7 years (IQR 28.8–43.4), with a median disease duration of 29.6 years (IQR 24.2–36.3). Most individuals had SMA type II (40.4%) or III (47.4%). Mean HFMSE changes from baseline were +1.00 (95% CI 0.05–1.95, p = 0.0100) at T1, +0.97 (95% CI 0.22–1.72, p = 0.0132) at T2, +1.78 (0.66–2.89, p = 0.0008) at T3, and +1.73 (0.49–2.97, p = 0.0049) at T4. Clinically meaningful improvements in motor function (≥3 points in HFMSE and/or ≥2 in RULM) were observed in 63.9% of patients at T4. Improvements were more pronounced in patients with higher baseline function, ambulatory status, or without a history of spinal surgery. Risdiplam was generally well tolerated, with predominantly mild and non-specific adverse events reported in 14.0% of patients.

**Interpretation:**

In this nationwide observational study in a real-world setting, adult patients with 5q-SMA demonstrated consistent and clinically meaningful functional improvements with risdiplam over time, particularly by 18 months and beyond. These findings support the long-term use of risdiplam in adults with SMA and help close a critical evidence gap in this underrepresented population.

**Funding:**

This study was financially supported by 10.13039/100004337F. Hoffmann-La Roche Ltd.

**Research in context**

**Evidence before this study**

We searched PubMed for articles using the terms “((spinal muscular atrophy) OR (SMA)) AND (risdiplam) AND (adult)” published between January 2019 and July 2025. Among 63 results, we identified eleven observational studies and two randomised controlled trials evaluating the efficacy of risdiplam in adult patients with 5q-SMA. However, most studies were limited by methodological constraints, small cohorts, or a primary focus on paediatric populations, thereby yielding inconsistent findings. The pivotal phase 3 randomised, placebo-controlled SUNFISH trial predominantly focused on children and adolescents, and included only 14 adults aged 18–25 years. In this subgroup, risdiplam showed no significant difference from placebo in the primary endpoint (change in the 32-item Motor Function Measure [MFM32]: −0.65 [95% CI −4.03 to 2.74]) or secondary outcomes, including the Hammersmith Functional Motor Scale Expanded (HFMSE; −0.73 [95% CI −3.78 to 2.32]) and the Revised Upper Limb Module (RULM; 1.74 [95% CI −1.06 to 4.53]) after 12 months. Similarly, the open-label JEWELFISH trial, which assessed risdiplam in previously treated patients only, included 34 adults aged 25–60 years and found no significant changes in MFM32, HFMSE, or RULM. Interpretation of the results, however, were limited by prior exposure to other disease-modifying therapies, precluding conclusions about the efficacy of risdiplam as monotherapy. Overall, the heterogeneity of endpoints, small sample sizes, and lack of statistical analysis rendered these findings on adult patients with 5q-SMA inconclusive.

**Added value of this study**

This is the first nationwide, multicentre study to assess the efficacy and safety of risdiplam in treatment-naïve adult patients with 5q-SMA. Statistically significant improvements were observed across multiple motor function measures (HFMSE, RULM, and Amyotrophic Sclerosis Functional Rating Scale Revised [ALSFRS-R]), supporting the therapeutic benefit of risdiplam in this adult population.

**Implications of all the available evidence**

Our findings provide robust real-world evidence for the efficacy of risdiplam in treatment-naïve adults with 5q-SMA. In contrast to prior studies with limited adult representation or inconsistent outcomes, this study demonstrates significant motor function improvements and supports the use of risdiplam as an effective treatment option for adult patients with 5q-SMA.

## Introduction

Spinal muscular atrophy (SMA) is a genetic motor neuron disease caused by homozygous deletions or, less commonly, pathogenic single-nucleotide point mutations in the *SMN1* gene.^1^ This results in a deficiency of the survival motor neuron (SMN) protein associated with predominant motor neuron degeneration in the spinal cord and brain stem. Clinically, SMA is characterised by progressive weakness and muscle wasting, ultimately resulting in tetraparesis and difficulties in swallowing and breathing.^2^, ^3^, ^4^, ^5^ The severity of SMA is influenced by the copy number of the paralogous *SMN2* gene, which produces a truncated SMN protein due to skipping of exon 7, with only a small proportion of functional SMN protein generated by each *SMN2* copy. Higher *SMN2* copy numbers can partially compensate for the *SMN1* deficiency and are associated with milder phenotypes.

Nusinersen, an antisense oligonucleotide, and risdiplam, a small molecule, both act as *SMN2*-specific splicing modifiers that promote the inclusion of exon 7 and lead to an increase in functional SMN protein levels.^6^ Despite the lack of robust efficacy data from randomised-controlled phase 3 trials in adult patients, both nusinersen and risdiplam were approved by the US Food and Drug Administration (FDA) and by the European Medicines Agency (EMA) for the treatment of SMA patients of all ages. Observational data on sufficiently large cohorts of adult SMA patients have been published and provide real-world evidence of clinical stabilisation or improvement with nusinersen treatment.^7^^,^^8^ For risdiplam, by contrast, evidence for efficacy in adults remains limited to case series, small single-centre studies (Appendix p 1),^9^, ^10^, ^11^, ^12^, ^13^, ^14^, ^15^, ^16^, ^17^, ^18^, ^19^ and the SUNFISH and JEWELFISH trials,^20^^,^^21^ which included only a small number of adults or, in case of the JEWELFISH trial, exclusively enrolled previously treated patients, thereby limiting the ability to draw robust conclusions on this subgroup.

In Austria, nusinersen was initially not reimbursed for adult patients due to the absence of adult data in pivotal trials and treatment was therefore deferred until 2020–2021, when the first adult data became available. By that time, risdiplam approval was imminent, and treatment selection was ultimately guided by shared decision-making between physicians and patients, and in some cases by reimbursement considerations. This unique situation resulted in a substantial proportion of treatment-naïve adults starting risdiplam, providing an opportunity to address the existing evidence gap. We therefore leveraged nationwide real-world data to conduct the first multicentre study on the efficacy and safety of risdiplam in treatment-naïve adults with 5q-associated SMA (5q-SMA).

## Methods

### Study design and patients

**Fig. 1:**
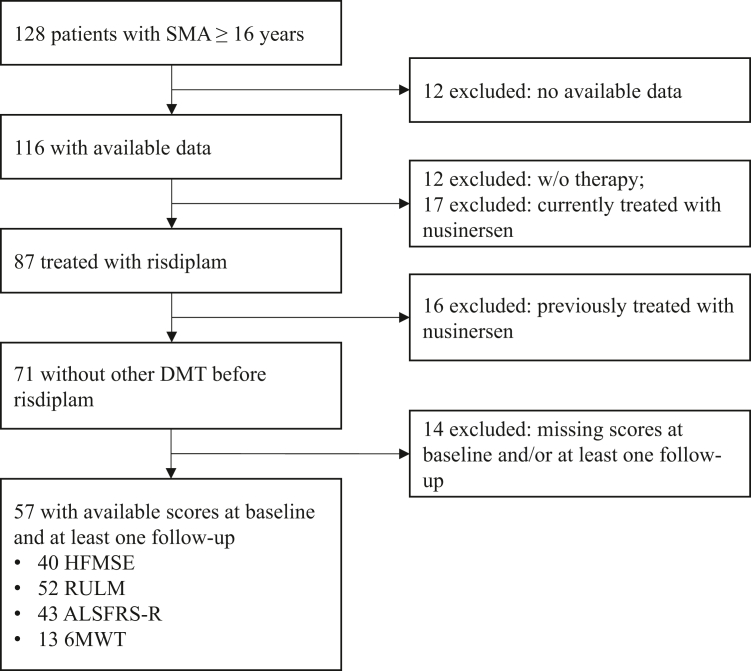
Inclusion flow chart. SMA, Spinal Muscular Atrophy; HFMSE, Hammersmith Functional Motor Scale Expanded; RULM, Revised Upper Limb Module; ALSFRS-R, Amyotrophic Lateral Sclerosis Functional Rating Scale Revised; 6MWT, 6 Minute Walk Test; DMT, Disease Modifying Therapy; w/o, without.

In this nationwide, observational cohort study, we included all Austrian adult patients (≥16 years treatment initiation) with genetically confirmed 5q-SMA who were previously therapy-naïve and received risdiplam for at least 3 months (Fig. 1). Patients were treated at eight neuromuscular centres between December 2020 and September 2024 (Departments of Neurology at the i) Medical University of Vienna, ii) Donaustadt Clinic Vienna, iii) Johannes Kepler University Linz, iv) Academic Teaching Hospital Wels-Grieskirchen, v) University Hospital Salzburg, vi) Medical University of Innsbruck, vii) Klagenfurt Clinic, and viii) Medical University of Graz). This study was approved by the Ethics committees of the Medical University of Vienna (No. 1800/2023), the City of Vienna (No. 24-014-VK), the Johannes Kepler University (No. 1066/2025), the State of Salzburg (No. 1032/2025), the Medical University of Innsbruck (No. 1059/2025), and the Medical University of Graz (No. 1075/2025). Informed consent was not required for this study.

### Procedures and outcomes

All patients treated with risdiplam were assessed for the availability of functional scores at baseline (T0) and at least one follow-up time point ≥3 months after treatment initiation. To evaluate changes from baseline in a pre-post comparison, the following time intervals for follow-up assessments were defined: 3–<6 months (T1), 6–<12 months (T2), 12–<18 months (T3), and ≥18 months (T4). The last available score within each predefined interval was used for the statistical analysis of pre-post differences. In addition, the scores from the patients’ overall last assessment, irrespective of predefined timepoints, were analysed in a pooled comparison. All available scores were included to plot individual patient trajectories.

The primary outcome was defined as the change from baseline in the Hammersmith Functional Motor Scale Expanded (HFMSE). The maximum HFMSE score is 66 points, and an improvement of ≥3 points was considered clinically meaningful. Secondary endpoints included changes from baseline in the Revised Upper Limb Module (RULM; maximum score 37 points, with a clinically meaningful improvement defined as ≥2 points), the 6-minute walk test (6MWT; distance walked in 6 minutes, in meters), and the revised Amyotrophic Lateral Sclerosis Functional Rating Scale (ALSFRS-R; maximum score 48 points). Scores documented until December 2024 were assessed by trained physiotherapists and, together with information on adverse events (AEs), extracted from the hospitals’ patient management databases.

### Statistical analysis

Descriptive statistics were performed using means with standard deviations (SDs) or medians with interquartile ranges (IQRs) and were calculated for the whole cohort and each predefined time interval. Pre-post changes from baseline to each pre-defined time point and to the last available score in HFMSE, RULM, 6MWT and ALSFRS-R were reported as means with 95% confidence intervals (CI) and assessed using the Wilcoxon signed rank test (Pratt's method). Individual patient trajectories in HFMSE, RULM, and ALSFRS-R were plotted relative to baseline scores and visualised using spaghetti plots, with LOESS smoothing lines used to estimate average change over time. Exploratory subgroup analyses of pre-post differences in HFMSE as the primary outcome were conducted for the latest time interval (T4) across the following parameters: SMA type (I/II and III/IV), HFMSE at baseline (<35 and ≥ 35 points), ambulatory status, and history of spinal surgery. Bland-Altman plots and linear regression (HFMSE [T4] = Intercept + b0∗HFMSE [T0]) were utilised to estimate the effect of baseline HFMSE on the change from baseline. Additionally, a linear mixed model analysis was performed with time, sex, age, and HFMSE at T0 as fixed effects, and patient as random effect. In a second analysis, a linear regression model was applied to assess potential centre effects, with outcome HFMSE at the last available assessment as the dependent variable and baseline HFMSE score included as an independent variable.

To evaluate the robustness of the primary endpoint to missing data, a sensitivity analysis was conducted using multiple imputation by chain equations with predictive mean matching. Baseline HFMSE and change from T0 to T4 were imputed, followed by passive calculation of HFMSE at T4. Imputations were conditional on sex, age, SMA type, ambulatory status, and HFMSE, and were constrained to the valid score range (0–66). Twenty imputation datasets were generated, and mean differences were pooled using Rubin's rules.

As no adjustment for multiple testing was performed, p-values are to be interpreted as explorative only. Statistical analyses and graphing were conducted using R (version 4.4.1, R Project for Statistical Computing), R Studio (version 2024.09.0 + 375, RStudio PBC), and Prism (version 10.5.0, GraphPad Software, Boston, MA, USA).

### Role of funding source

This study was financially supported by F. Hoffmann-La Roche Ltd. The authors were solely responsible for the study design, data collection, data analysis, data interpretation, writing of the manuscript, and the decision to submit the manuscript for publication. The funder was not involved in any of these aspects.

## Results

A total of 128 adult patients with 5q-SMA were screened for eligibility (Fig. 1). Twelve patients were excluded due to missing data, 12 had not received any treatment, and 17 were receiving nusinersen. Among the remaining 87 patients, 71 were treatment-naïve at the time of risdiplam initiation. Baseline and at least one follow-up assessment were available for 57 patients (80.3%; 32 [56.1%] female), who were included in the outcome analyses (Table 1). In this cohort, the median age at treatment initiation was 35.7 years (IQR 28.8–43.4), and the median disease duration was 29.6 years (IQR 24.2–36.3). Most patients had three *SMN2* copies (54.4%) and were classified as SMA type II (40.4%) or III (47.4%). The proportion of ambulatory patients was 22.8%. HFMSE scores were available for 40 patients (70.2%), RULM scores for 52 (91.2%), ALSFRS-R for 43 (75.4%) and 6MWT data for 13 patients (22.8%). In the HFMSE analyses, 6.7% and 42.3% of patients had baseline scores >60 and < 5 points, respectively, indicating potential ceiling and floor effects.^22^ Similarly, in the RULM analyses, baseline scores >35 and < 10 were observed in 16.7% and 47.5% of patients, respectively.

**Table 1:** Baseline characteristics and demographics of the whole cohort and based on the predefined timepoints.

	Whole cohort (n = 57)	Included in T1 analysis (n = 41)	Included in T2 analysis (n = 44)	Included in T3 analysis (n = 38)	Included in T4 analysis (n = 36)
Female	32 (56.1%)	23 (56.1%)	23 (52.3%)	21 (55.3%)	19 (52.8%)
Age at first therapy, years	35.7 (28.8–43.4)	37.2 (29.0–46.9)	35.8 (29.7–45.9)	35.6 (29.6–41.3)	35.0 (29.5–41.7)
Treatment time until last examination, months	21.0 (12.5–24.0)	22.0 (12.0–24.0)	22.0 (14.3–24.0)	23.0 (18.0–24.3)	23.0 (22.0–25.8)
Disease duration before treatment, years	29.6 (24.2–36.3)	28.5 (22.3–36.3)	30.1 (24.5–36.3)	30.1 (23.9–36.3)	28.8 (24.0–35.2)
Patients with available information	43 (75.4%)	31 (75.6%)	35 (79.5%)	27 (71.1%)	28 (77.8%)
Spinal muscle atrophy type					
Type I	2 (3.5%)	1 (2.4%)	2 (4.5%)	2 (5.3%)	2 (5.6%)
Type II	23 (40.4%)	15 (36.6%)	18 (40.9%)	13 (34.2%)	14 (38.9%)
Type III	27 (47.4%)	20 (48.8%)	21 (47.7%)	21 (55.3%)	18 (50.0%)
Type IV	4 (7.0%)	4 (9.8%)	2 (2.3%)	2 (5.3%)	2 (5.6%)
Unknown	1 (1.8%)	1 (2.4%)	1 (2.3%)	0 (0.0%)	0 (0.0%)
*SMN2* copies					
1	2 (3.5%)	1 (2.4%)	1 (2.3%)	2 (5.3%)	1 (2.8%)
2	4 (7.0%)	3 (7.3%)	4 (9.1%)	2 (5.3%)	2 (5.6%)
3	31 (54.4%)	21 (51.2%)	24 (54.5%)	21 (55.3%)	21 (58.3%)
4	17 (29.8%)	14 (34.1%)	15 (34.1%)	10 (26.3%)	10 (27.8%)
Unknown	3 (5.3%)	2 (4.9%)	0 (0.0%)	3 (7.9%)	2 (5.6%)
Ambulatory status					
Ambulatory	13 (22.8%)	10 (24.4%)	10 (22.7%)	10 (26.3%)	8 (22.2%)
Non-ambulatory	44 (77.2%)	31 (75.6%)	34 (77.3%)	28 (73.7%)	28 (77.8%)
Spinal surgery					
No	27 (47.4%)	17 (41.5%)	18 (40.9%)	20 (52.6%)	18 (50.0%)
Yes	16 (28.1%)	12 (29.3%)	14 (31.8%)	10 (26.3%)	9 (25.0%)
Unknown	14 (24.6%)	12 (29.3%)	12 (27.3%)	8 (21.1%)	9 (25.0%)
Baseline HFMSE					
≥35	13 (22.8%)	9 (22.0%)	10 (22.7%)	10 (26.3%)	8 (22.2%)
<35	29 (50.9%)	23 (56.1%)	21 (47.7%)	19 (50.0%)	21 (58.3%)
Unknown	15 (26.3%)	9 (22.0%)	13 (29.5%)	9 (23.7%)	7 (19.4%)
No. of patients included in HFMSE analysis	40 (70.2%)	30 (73.2%)	30 (68.2%)	27 (71.1%)	26 (72.2%)
Treatment time until last HFMSE, months	19.5 (12.0–24.8)	–	–	–	–
No. of patients included in RULM analysis	52 (91.2%)	36 (87.8%)	40 (90.9%)	35 (92.1%)	34 (94.4%)
Treatment time until last RULM, months	22.0 (13.3–24.0)	–	–	–	–
No. of patients included in ALSFRS-R analysis	43 (75.4%)	27 (65.9%)	32 (72.7%)	29 (76.3%)	28 (77.8%)
Treatment time until last ALSFRS-R, months	22.0 (12.0–25.0)	–	–	–	–
No. of patients included in 6MWT analysis	13 (22.8%)	9 (22.0%)	10 (22.7%)	10 (26.3%)	7 (19.4%)
Treatment time until last 6MWT, months	18.0 (11.0–24.0)	–	–	–	–

Data are n (%) or median (IQR). HFMSE, Hammersmith Functional Motor Scale Expanded; RULM, Revised Upper Limb Module; ALSFRS-R, Amyotrophic Lateral Sclerosis Functional Rating Scale Revised; 6MWT, 6-Minute Walk Test.

**Fig. 2:**
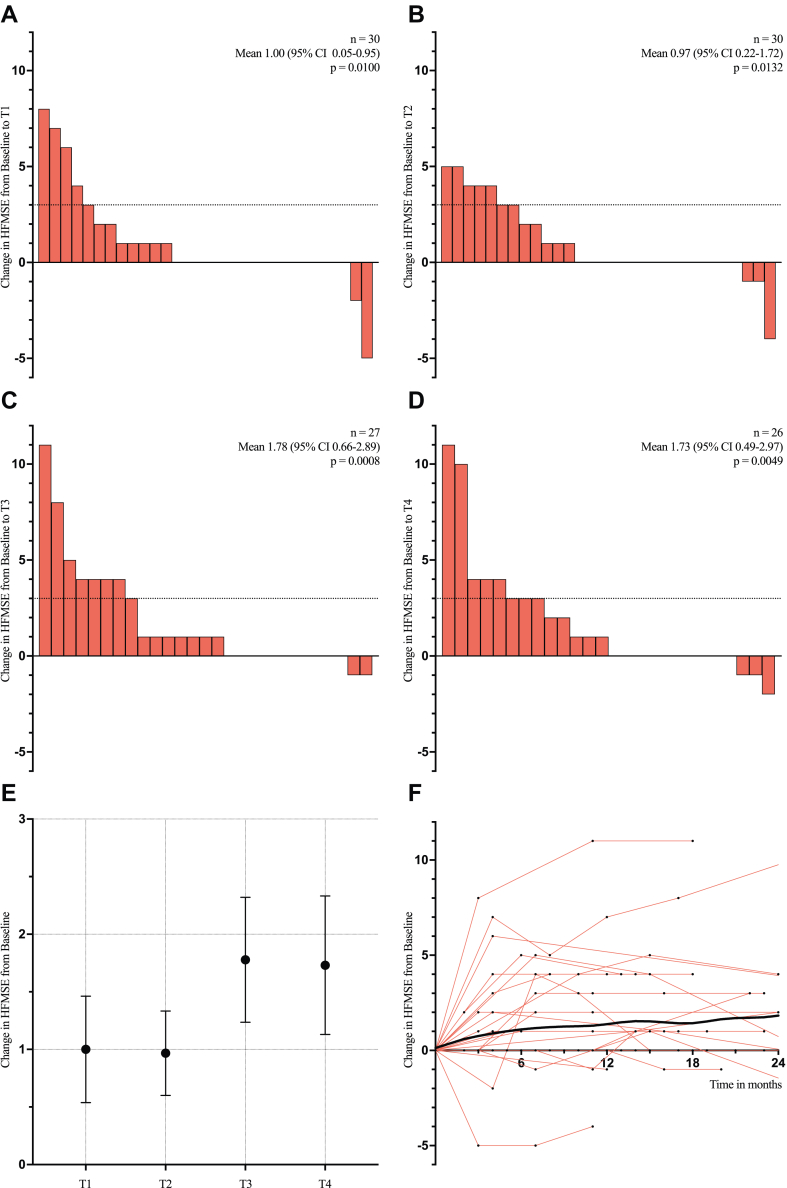
**(A**–**D)** Changes in HFMSE from baseline at the predefined timepoints, with bars representing individual patients. Dotted lines mark the threshold for clinically meaningful improvement. Reported p-values correspond to the Wilcoxon signed rank test. **(E)** Mean pre-post changes (with standard error) in HFMSE at each predefined timepoint. **(F)** Individual HFMSE trajectories (red) after treatment initiation, with baseline scores normalised to 0. The LOESS smoothing line (black) estimates average HFMSE change over time. HFMSE, Hammersmith Functional Motor Scale Expanded; 95% CI, 95% Confidence Interval; LOESS, Locally Estimated Scatterplot Smoothing.

The mean change in HFMSE compared to baseline ranged from +1.00 (95% CI 0.05–1.95, p = 0.0100) at T1 and +0.97 (95% CI 0.22–1.72, p = 0.0132) at T2 to +1.78 (95% CI 0.66–2.89, p = 0.0008) at T3 and +1.73 (95% CI 0.49–2.97, p = 0.0049) at T4, respectively (Fig. 2A–E, Table 2). The mean change between baseline and the last available score was +1.23 (95% CI 0.35–2.10, p = 0.0037). The proportion of patients achieving a clinically meaningful improvement of ≥3 points rose steadily from 16.7% at T1 to 30.8% at T4 (Appendix p 2). The average trajectory of HFMSE from baseline, estimated by LOESS, was consistently positive (Fig. 2F, Appendix p 3).

**Table 2:** Mean changes in HFMSE, RULM, ALSFRS-R, and 6MWT versus baseline.

	n	Mean change from baseline (95% CI)	p-value
**HFMSE**			
T1	30	1.00 (0.05–1.95)	0.0100
T2	30	0.97 (0.22–1.72)	0.0132
T3	27	1.78 (0.66–2.89)	0.0008
T4	26	1.73 (0.49–2.97)	0.0049
At last assessment	40	1.23 (0.35–2.10)	0.0037
**RULM**			
T1	36	1.67 (0.76–2.58)	<0.0001
T2	40	2.05 (0.98–3.12)	0.0001
T3	35	2.51 (1.48–3.55)	<0.0001
T4	34	2.76 (1.59–3.94)	<0.0001
At last assessment	52	2.44 (1.51–3.37)	<0.0001
**ALSFRS-R**			
T1	27	0.41 (−0.49–1.29)	0.6636
T2	32	0.78 (0.14–1.43)	0.0365
T3	29	0.90 (0.06–1.73)	0.1099
T4	28	1.36 (0.50–2.22)	0.0076
At last assessment	43	1.02 (0.35–1.70)	0.0200
**6MWT**			
T1	9	52.7 (2.0–103.3)	0.0039
T2	10	44.7 (−10.6–100.0)	0.1172
T3	10	19.4 (−53.4–92.2)	0.7891
T4	7	47.6 (−59.0–154.1)	0.4688
At last assessment	13	36.5 (−16.9–89.8)	0.1978

95% CI, 95% Confidence Interval; HFMSE, Hammersmith Functional Motor Scale Expanded; RULM, Revised Upper Limb Module; ALSFRS-R, Amyotrophic Lateral Sclerosis Functional Rating Scale Revised; 6MWT, 6-Minute Walk Test.

**Fig. 3:**
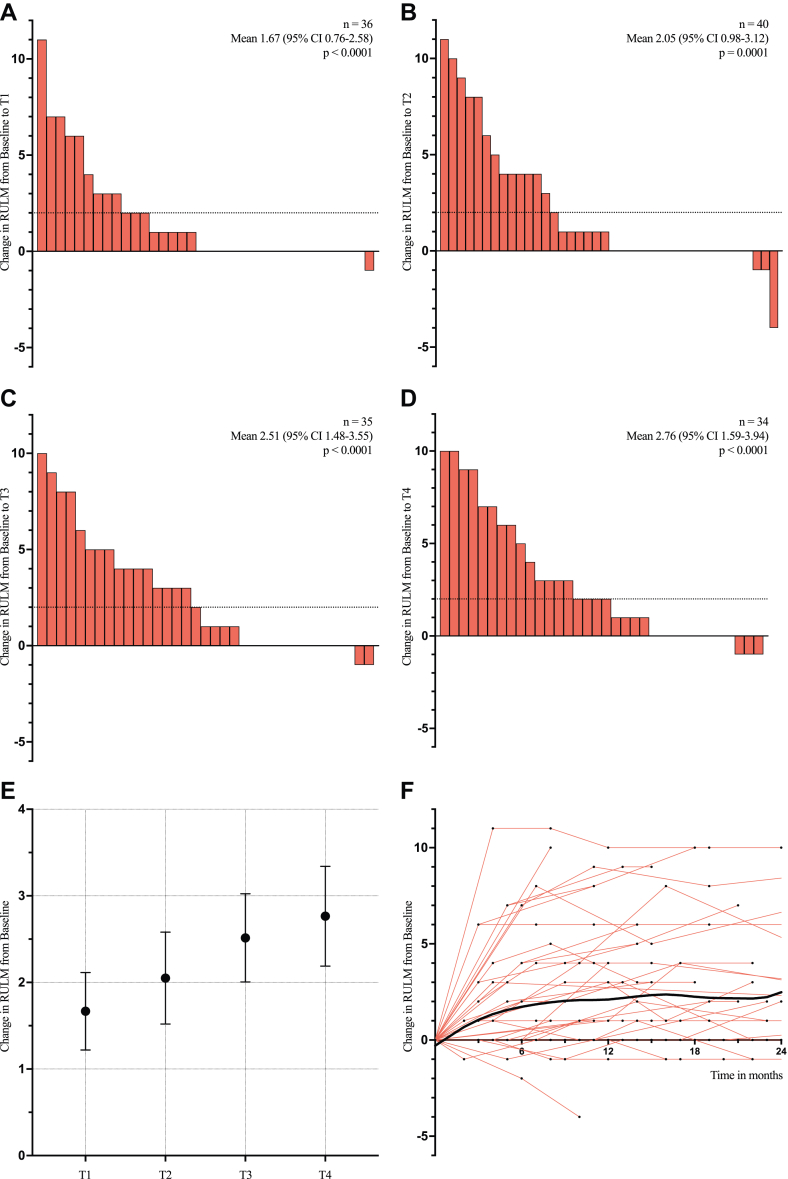
**(A**–**D)** Changes in RULM from baseline at the predefined timepoints, with bars representing individual patients. Dotted lines mark the threshold for clinically meaningful improvement. Reported p-values correspond to the Wilcoxon signed rank test. **(E)** Mean pre-post changes (with standard error) in RULM at each predefined timepoint. **(F)** Individual RULM trajectories (red) after treatment initiation, with baseline scores normalised to 0. The LOESS smoothing line (black) estimates average RULM change over time. RULM, Revised Upper Limb Module; 95% CI, 95% Confidence Interval; LOESS, Locally Estimated Scatterplot Smoothing.

Improvements in upper limb function assessed by RULM were more pronounced than those observed in HFMSE, with the mean changes rising from +1.67 (95% CI 0.76–2.58, p < 0.0001) at T1 to +2.76 (95% CI 1.59–3.94, p < 0.0001) at T4 (Fig. 3A–E, Table 2). Overall, the mean improvement from baseline to the last available assessment was +2.44 (95% CI 1.51–3.37, p < 0.0001). The proportion of patients with a clinically meaningful improvement of ≥2 points in RULM increased from 33.3% at T1 to 52.9% at T4 (Appendix p 4). A clinically meaningful improvement in at least one motor function score (improvement of ≥3 points in HFMSE or ≥2 points in RULM) was observed in 31.7% at T1, increasing to 43.2% at T2, 55.3% at T3, and 63.9% at T4. Mean changes in ALSFRS-R scores also improved over time, with mean changes increasing from +0.41 (95% CI −0.49 to 1.29, p = 0.6636) at T1 to +1.36 (95% CI 0.50–2.22, p = 0.0076) at T4 (Table 2, Appendix pp 5 and 6). Interpretation of 6MWT results was limited by the small number of patients with available data (ranging from 7 to 10 patients across time intervals; Appendix p 7). However, improvements in walking distance were observed at all time points, ranging from 52.7 m at T1 to 47.6 m at T4. The average trajectories of RULM and ALSFRS-R, estimated by LOESS, remained consistently positive (Fig. 3F, Appendix pp 3, 6).

Exploratory subgroup analyses of HFMSE changes from baseline revealed that improvements were generally greater in subgroups reflecting higher baseline functional status (Table 3). Patients with SMA types III or IV experienced a mean improvement of +2.50 (95% CI 0.54–4.46, p = 0.0132) at T4, whereas those with SMA type I or II improved by +0.50 (95% CI −0.20 to 1.20, p = 0.2500). Among patients grouped by baseline HFMSE scores, improvements at T4 were comparable between groups, with a mean change of +1.67 (95% CI −0.07 to 3.41, p = 0.0488) in patients with baseline HFMSE scores <35, and +1.90 (95% CI 0.24–3.51, p = 0.0625) in those with baseline score ≥35. Further analysis by Bland-Altman plot and linear regression revealed that the baseline HFMSE at T0 was a strong predictor of the outcome HFMSE at T4 (slope estimate 1.06, 95% CI 1.00–1.12, p < 0.0001; Appendix p 8). This finding was confirmed by mixed model analysis, where only baseline HFMSE and time emerged as significant predictors of HFMSE change (estimate 1.01, 95% CI 0.98–1.04, and 0.08, 95% CI 0.04–0.11, respectively; p < 0.0001). Moreover, non-ambulatory patients improved by +1.39 (95% CI 0.02–2.76, p = 0.0195), while ambulatory patients experienced a higher improvement (+2.05, 95% CI −0.66 to 5.66, p = 0.1094), which, however, was statistically not significant, likely due to the smaller sample size in this subgroup. Patients without a history of spinal surgery had a larger, although again not statistically significant, mean improvement at T4 (+2.46, 95% CI −0.32 to 5.23, p = 0.0625) compared to those with spinal surgery (+1.14, 95% CI −0.41 to 2.69, p = 0.2500). Consistent with these findings, LOESS curves showed higher average trajectories over time in subgroups presenting with a higher functional status at baseline (Appendix p 9). Importantly, HFMSE outcome was not significantly influenced by treatment centre.

**Table 3:** Exploratory subgroup analyses of HFMSE changes at T4.

	n	Mean change from baseline (95% CI)	p-value
**Spinal muscular atrophy type**			
I/II	10	0.50 (−0.20–1.20)	0.2500
III/IV	16	2.50 (0.54–4.46)	0.0132
**HFMSE at baseline**			
<35	18	1.67 (−0.07–3.41)	0.0488
≥35	8	1.90 (0.24–3.51)	0.0625
**Ambulatory**			
No	18	1.39 (0.02–2.76)	0.0195
Yes	8	2.05 (−0.66–5.66)	0.1094
**Spinal surgery**			
Yes	7	1.14 (−0.41–2.69)	0.2500
No	11	2.46 (−0.32–5.23)	0.0625

95% CI, 95% Confidence Interval; HFMSE, Hammersmith Functional Motor Scale Expanded.

Sensitivity analysis confirmed the robustness of these findings, yielding a pooled estimated mean HFMSE change from baseline to T4 of +1.96 (95% CI 0.70–3.21, p = 0.0042). Per-imputation Wilcoxon signed-rank tests consistently showed low p-values ranging between <0.0001 and 0.0005.

AEs were reported in eight patients (14.0%) (Appendix p 10). Sensitive or dry skin and gastrointestinal symptoms were reported by two and three patients, respectively. Nausea, headache, and concentrated urine were each reported by one patient. One patient showed slightly elevated amylase and lipase after treatment initiation, which remained stable at <2× the upper limit of normal on follow-up and were not associated with symptoms of alternative pathology. Three events including a bone fracture, an acute cardiac event, and a dental infection were reported, but deemed unrelated to risdiplam by the investigators.

## Discussion

In this real-world cohort of adult patients with 5q-SMA, treatment with risdiplam was associated with consistent motor function improvement in most patients over a median follow-up time of 21 months, with the trajectory of change remaining steadily positive throughout the observation period. Clinically meaningful improvements were measured in 30.8% of patients based on the HFMSE score and in 52.9% of patients based on the RULM score. Exploratory subgroup analyses indicated that patients with higher baseline functional status tended to experience greater mean improvement in HFMSE scores. These findings are consistent with previous observations in nusinersen-treated adults, where better preserved motor function at baseline was associated with more pronounced treatment responses.^7^ AEs were reported in 14% of the cohort without any treatment discontinuations, indicating overall good tolerability of risdiplam in adult patients.

Previous data on risdiplam in adult patients have been limited to case series and small single-centre studies with notable methodological constraints and conflicting results. A retrospective study in a cohort of 14 adult patients with SMA reported inconsistent improvements in HFMSE and RULM scores across different follow-up assessments, with limited statistical power and prior nusinersen exposure in one patient.^17^ Another recently published retrospective study including 18 patients, 16 of whom were non-ambulatory, reported significant improvement in the Motor Function Measure-32 (MFM32) after 24 months, whereas RULM findings were inconsistent, with significant improvement at 12 months but not at 24 months.^19^ A rigorously designed prospective study applied fixed follow-up intervals and standardised assessments in 25 non-ambulatory adults, reporting a modest increase in RULM but no change in HFMSE scores. However, this study was also limited by a small sample size with only 19 patients evaluated at the 12-month time point, and was not representative of the broader adult SMA population due to the recruitment of purely non-ambulatory patients from a single site.^11^ Another study in 18 adult patients found significant improvements in the HFMSE and the Children's Hospital of Philadelphia Infant Test of Neuromuscular Disorders at 10 months after treatment initiation but no significant RULM change. However, this study primarily examined correlations between electrophysiological and clinical outcomes and was also constrained by small sample size.^13^ A separate study on a cohort of 31 treatment-naïve adult SMA patients reported that 22.6% improved in at least one motor function measure, yet statistical significance was not reported and raw data were insufficiently provided to enable further analyses.^16^ Published case series included between four to nine patients and were further limited by heterogeneity in follow-up duration and conflicting findings, providing only anecdotal evidence for the effects of risdiplam.^10^^,^^12^^,^^14^^,^^18^

The phase 3 placebo-controlled SUNFISH trial remains the most pivotal study evaluating the efficacy of risdiplam in paediatric and adult patients with type II and III SMA; however, only 14 adults, aged 18–25 years, were included in the treatment arm.^20^ After 12 months, improvements in MFM32 and RULM were observed in the risdiplam-treated group compared to placebo, but not in HFMSE. Adult patients improved only in RULM, albeit not significantly (estimate +1.74 in a mixed model repeated measure analysis; compared to +1.67 at T1, +2.05 at T2, +2.51 at T3, and +2.76 at T4 in our study). In the open-label extension (OLE) study evaluating two-year efficacy of risdiplam in 10 adults, changes from baseline in HFMSE and RULM closely mirrored our findings.^23^ Mean improvements of 1.4 and 2.1 at 18 months and 2.2 and 2.8 at 24 months, respectively, were reported, compared to 1.73 and 2.76 at T4 (with a median treatment time of 23 months) in our study. The proportions of patients with clinically meaningful improvements were also comparable (HFMSE: 30.8% versus 39% at 18 months and 45% at 24 months, RULM: 52.9% versus 50% at 18 months and 52% at 24 months). Although both the SUNFISH study and its OLE primarily focused on paediatric populations, their results support and corroborate the findings in our cohort.

Our study addresses many of the limitations of these studies and constitutes the largest observational cohort of adult patients with SMA treated with risdiplam to date. A particular strength lies in the inclusion of a treatment-naïve population, allowing for a more accurate evaluation of the therapeutic effect of risdiplam without confounding from prior exposure to nusinersen. The nationwide, multicentre design encompassing all major neuromuscular centres in Austria ensures the broad applicability of our findings. Standardised, physiotherapist-administered assessments using multiple validated functional outcome measures ensured consistency and robustness of efficacy data across centres. Furthermore, the extended observation period enabled us to characterise both the onset and persistence of motor function responses.

However, there are also limitations inherent to the observational study design that need to be acknowledged. While the overall size of the cohort was substantial, sample sizes within specific subgroups such as those grouped by baseline HFMSE scores were limited, thereby restricting statistical power of subgroup analyses and increasing the risk of type II errors. These analyses should therefore be considered as exploratory. To address this limitation, we analysed individual trajectories of motor function over time, which improved the ability to detect treatment-related changes even when subgroup sizes at single time points were small. Improvements from baseline were consistently more pronounced in patients with higher baseline function. These findings are also in line with the SUNFISH trial, as well as the observational cohort study on the efficacy nusinersen in adult patients with SMA.^7^^,^^20^ Another limitation is the absence of a control group, inherent to the observational nature of the study. This design raises the possibility that patients receiving risdiplam may have benefited from more intensive monitoring or supportive care, potentially influencing outcomes independently of the therapeutic effect of risdiplam. Nevertheless, the Austrian health care system provides universal and equal access to care, that is essentially free, thereby limiting bias in patient recruitment. In our study, all patients had access to standard of care prior to treatment initiation, and the multicentre structure with standardised assessments aimed to minimise such confounding factors. Moreover, in the presence of already approved and widely available disease-modifying therapies such as nusinersen, conducting a controlled study by withholding treatment is neither ethically justifiable nor feasible in adult patients with SMA. Our findings should therefore be interpreted within the context of routine clinical practice.

A recognised challenge in the assessment of motor function in SMA is the presence of ceiling and floor effects in commonly used outcome measures, particularly the HFMSE and RULM.^8^^,^^24^^,^^25^ Ceiling effects occur when patients score near the upper end of a scale, limiting the ability to detect further improvement, while floor effects can obscure subtle deteriorations or improvements in more severely affected individuals. Based on established thresholds, ceiling effects are likely to be found in patients with HFMSE scores >60 or RULM scores >35, and floor effects in those with HFMSE scores <5 or RULM scores <10.^22^ In our cohort, floor effects were likely to be observed in more patients than ceiling effects, with up to 42.5% of patients scoring below an HFMSE of 5 and up to 47.5% scoring below a RULM of 10 at different time points, reflecting the challenge of capturing motor changes in more severely affected adults with SMA in a real-world setting. To address the limitations associated with these effects, we applied a multimodal assessment strategy employing the RULM to evaluate upper limb function in non-ambulatory patients and the 6MWT to measure ambulatory capacity, as successfully implemented in an observational study on nusinersen.^7^ Although the ALSFRS-R was not developed specifically for SMA and is limited by non-linearity and low sensitivity to minor clinical changes, its validity has been demonstrated in adult patients with SMA with no concomitant ceiling or floor effects.^22^ Notably, ALSFRS-R changes have been shown to correlate with HFMSE outcomes in patients treated with nusinersen and were particularly sensitive to clinical improvements in ambulatory patients,^22^^,^^26^ even to a greater extent than the 6MWT.^22^ This was especially valuable in the context of our study, given the limited number of available 6MWT assessments. Overall, our multimodal approach enabled a more comprehensive evaluation across the full spectrum of disease severity and enhanced the probability to detect treatment-related changes even in cases where one scale alone may have lacked sensitivity. Future studies and clinical care structures could further enhance assessment by incorporating patient-reported outcome measures such as the SMA Independence scale, which, similar to the ALSFRS-R, evaluates activities of daily living and specifically captures SMA-related functional limitations in both ambulatory and non-ambulatory patients.^27^^,^^28^

In conclusion, our study provides real-world evidence supporting the efficacy and safety of risdiplam in adult patients with 5q-SMA. Future studies should focus on long-term outcomes, the elucidation of predictors of treatment response and the examination of the effect of risdiplam on non-motor symptoms, which have been increasingly recognised in SMA.^16^^,^^29^ Moreover, comparative studies between risdiplam and nusinersen will be essential to determine the most appropriate therapeutic approach for different SMA subgroups.

## Contributors

OK planned and coordinated the study, managed, analysed and interpreted the data, and wrote the manuscript. BF, VAG and NL were responsible for local data collection. TW, AE and ES were involved in analysis of the data and critically revised the manuscript. ME, GZ, JR, MK, CK, TAG, VG, JW, AH, VEK, CGH, JT, SG, PM, DL, MR, AW, MGT, FK, SM, GB, MB, FZ, RT, CE, SQ, and WL were responsible for local data collection and critically revised the manuscript. HC planned, coordinated and supervised the study, interpreted the data, was responsible for local data collection, and wrote the manuscript. OK and HC accessed and verified the data. All authors had full access to the data. All authors have agreed with the content of this manuscript and gave their consent for publication.

## Data sharing statement

No patient data or study related documents are shared within this paper. Reasonable requests from qualified investigators will be considered by the corresponding author in accordance with applicable privacy regulations.

## Declaration of interests

OK received support to attend scientific conferences or meetings from Roche and Biogen and speaker's honoraria from Biogen. ME received honoraria for lectures from Roche and for advisory boards from Roche, ScholarRock and Biogen, as well as support to attend a scientific conference from Roche. MK received travel support from Roche to attend scientific conferences. CK received support to attend scientific meetings as well as travel fees from Roche. TAG received travel compensation and compensation to attend scientific meetings from Biogen and Roche. CGH received support to attend scientific meetings from Biogen, Amicus Therapeutics, and speakers's honoraria from Grünenthal. JT received travel support from Roche to attend scientific conferences. SG received fees for advisory boards from Roche. PM received compensation from Roche to attend an advisory board, and support to attend a scientific meeting from Lundbeck Pharma. MR received consultation fees from Roche. AW received speaker's honoraria as well as conference and travel fees from Roche. MGT received advisory board fees and speaker's honoraria from Novartis and Roche, and support to attend scientific conferences from Roche. SM received consultation fees, speaker's or advisory board honoraria from Novartis, Roche and ScholarRock, and support for attending scientific conferences from Roche. GB received fees for advisory boards and consultations, and support to attend scientific conferences from Novartis, Biogen and Roche. MB received compensation for advisory boards and speaker's honoraria from Novartis, Biogen and Roche and travel compensation to meetings from Roche. RT received support to attend scientific meetings from Angelini, AstraZeneca, Janssen-Cilag, Merz, Pfizer, Sanofi, Biogen and Roche, speaker's honoraria from Abbvie, Alexion, ArgenX, BMS, Daiichi-Sankyo and Teva-ratiopharm. as well as advisory board fees from Alexion, ArgenX, Grünenthal, Janssen-Cilag, Sanofi, UCB, Biogen, Novartis and Roche. CE received fees for advisory boards from Roche and Biogen, and owns stock options from Roche. WL received fees for advisory boards or speaker's honoraria from Novartis, Biogen and Roche, and payments for patents from Roche and Novartis. WL also owns stock options from Novartis. HC received fees for advisory boards and speaker's honoraria from Biogen, Novartis and Roche, and also received funding for research projects from these companies. All other authors declared no conflicts of interest.
