# Supplementary material for: Efficacy and safety of risdiplam in adults with 5q-associated spinal muscular atrophy: a nationwide observational cohort study in Austria
Source: eClinicalMedicine. 2025 Sep 26;88:103536. doi: 10.1016/j.eclinm.2025.103536 (PMC12572788; doi:10.1016/j.eclinm.2025.103536)
Supplement: Supplementary Appendix [file mmc1.docx]

**Table** List of previous studies on the efficacy of risdiplam in adult patients, which included treatment-naïve patients.

| **Author and publication year** | **Proportion of treatment-naïve patients prior to risdiplam** | **Clinical and patient reported assessments** | **Follow-up time** | **Summary of treatment effects** |
| --- | --- | --- | --- | --- |
| Sitas et al. (2024)^1^ | 31/31 | RHS, RULM, 6MWT, JFLS, INQoL | 16 and 30 months, respectively (median) | Clinically meaningful improvement in 22.6% of patients in at least one motor function score (RHS or RULM). 60% reported improvement in at least one item on the JFLS. However, mean changes from baseline were not statistically analysed. |
| Severa et al. (2024)^2^ | 4/6 | MFM32, HFMSE | 16 months (mean) | Three treatment-naïve patients experienced clinically meaningful improvement in MFM32, but none improved in HFMSE. |
| McCluskey et al. (2023)^3^ | 6/6 | RULM, EK2, ESS, QOLM, FVC, FEV1 | 9 months (predefined timepoint; RULM assessed after 6 months) | Two patients experienced meaningful improvement in RULM. Overall, mean improvement in QOLM and mean worsening in EK2 were significant, but changes from baseline in other scores were not significant. |
| Kessler et al. (2024)^4^ | 18/18 | HFMSE, RULM, CHOP-INTEND | 10 months (predefined timepoint) | Focus on the correlation between electrophysiological and clinical outcome measures. Changes from baseline in HFMSE and CHOP INTEND improved significantly, but not in RULM. |
| Nungo Garzon et al. (2023)^5^ | 4/6 | RULM, EK2, ALSFRS-R, C-GIC, P-GIC, GAS, BMI, FVC | 12 months (predefined timepoint) | Clinically meaningful improvement was observed in at least one score in all patients, with treatment-naïve patient showing meaningful improvement in the upper limb function. |
| Gavriilaki et al. (2024)^6^ | 13/14 | MFM32, HFMSE, RULM  SMAFRS, FVC | 25 months (median) | HFMSE, RULM and MFM32, but not SMAFRS, improved significantly at last assessment. However, only up to 8 patients were included at different time points, in which mean changes were not consistently significant. |
| Brakemeier et al. (2024)^7^ | 25/25 | HFMSE, RULM, bulbar domain of ALSFRS-R, SSQ | Up to 12 months | This study focussed on the effect of risdiplam on bulbar function. Significant improvement was observed in RULM and SSQ after 12 months, but not in HFMSE or in bulbar function assessed by the ALSFRS-R. |
| Bjelika et al. (2024)^8^ | 9/14 | HFMSE, RULM | Up to 20 months | Clinically meaningful improvement based on HFMSE was not detected. In three (33·3%) and five patients (55·6%), who were treatment-naïve prior to risdiplam, upper limb function improved or deteriorated, respectively. |
| Iterbeke et Claeys (2025)^9^ | 18/18 | MFM32, MRC, RULM, FOIS, NdSSS, FVC, PEF, SF-36, FSS, SMAIS, SSQ, grip and pinch strength | 24 months (predefined timepoint) | Focus primarily on non-ambulatory (16/18) patients, improvements observed in MFM-32 after 24 months and RULM after 12 months but not after 24 months; no consistent improvements in any of the other motor function scores. |

ALSFRS-R=Amyotrophic Lateral Sclerosis Functional Rating Scale Revised. BMI=Body Mass Index. C-GIC=Clinical Global Impression of Change. CHOP-INTEND= Children’s Hospital Of Philadelphia Infant Test of Neuromuscular Disorders. EK2=Egen Klassifikation 2. ESS=Epworth Sleepiness Scale. FEV1=Forced Expiratory Volume. FOIS=Functional Oral Intake Scale. FVC=Forced Vital Capacity. GAS=Global Attainment Scale. HFMSE=Hammersmith Functional Motor Scale Expanded. INQoL=Individualised Neuromuscular Quality of Life Questionnaire. JFLS=Jaw Functional Limitation Scale. MFM32=32-item Motor Function Measure. MRC=Medical Research Council Sum Score. NdSSS=Neuromuscular Disease Swallowing Status Scale. PEF=Peak Expiratory Flow. P-GIC=Patient Global Impression of Change. QOLM=Quality of Life Measure for people with slowly progressive and genetic neuromuscular disease. RHS=Revised Hammersmith Score. RULM=Revised Upper Limb Module. SF-36=Short-form 36 Health Survey. SMAFRS=Spinal Muscular Atrophy Functional Rating Scale. SMAIS=Spinal Muscular Atrophy Independence Scale. SSQ=Sydney Swallow Questionnaire. 6MWT=6-Minute Walk Test.

**Table** Characteristics of patients included in the HFMSE analysis.

|  | **Included in T1 analysis (n=30)** | **Included in T2 analysis (n=30)** | **Included in T3 analysis (n=27)** | **Included in T4 analysis (n=26)** |
| --- | --- | --- | --- | --- |
| Female | 17 (56·7%) | 14 (46·7%) | 15 (55·6%) | 11 (42·3%) |
| Age at first therapy, years | 35·8 (28·3-47·6) | 36·6 (30·8-47·4) | 35·6 (29·5-41·8) | 35·0 (29·6-43·8) |
| Treatment time until last examination, months | 21·5 (11·0-24·3) | 22·5 (17·8-24·3) | 23·0 (18·0-25·0) | 23·0 (19·8-25·3) |
| Disease duration before treatment, years  Patients with available  information | 25·7 (22·2-34·0)  23 (76·7%) | 30·1 (24·2-35·4)  23 (76·7%) | 29·0 (23·7-35·4)  19 (70·4%) | 26·7 (22·6-35·2)  20 (76·9%) |
| Time from treatment start to analysis, months | 4·0 (3·0-4·3) | 9·0 (7·0-11·0) | 15·0 (14·0-16·0) | 23·5 (19·0-26·5) |
| Spinal muscular atrophy type  Type I  Type II  Type III  Type IV  Unknown | 0 (0·0%)  10 (33·3%)  15 (50·0%)  4 (13·3%)  1 (3·3%) | 1 (3·3%)  9 (30·0%)  17 (56·7%)  2 (6·7%)  1 (3·3%) | 1 (3·7%)  8 (29·6%)  16 (59·3%)  2 (7·4%)  0 (0·0%) | 1 (3·8%)  9 (34·6%)  14 (53·8%)  2 (7·7%)  0 (0·0%) |
| *SMN2* copies  1  2  3  4  Unknown | 1 (3·3%)  3 (10·0%)  13 (43·3%)  12 (40·0%)  1 (3·3%) | 1 (3·3%)  4 (13·3%)  14 (46·7%)  11 (36·7%)  0 (0·0%) | 1 (3·7%)  2 (7·4%)  13 (48·1%)  9 (33·3%)  2 (7·4%) | 0 (0·0%)  1 (3·8%)  15 (57·7%)  9 (34·6%)  1 (3·8%) |
| Ambulatory status  Ambulatory  Non-ambulatory | 10 (33·3%)  20 (66·7%) | 10 (33·3%)  20 (66·7%) | 10 (37·0%)  17 (63·0%) | 8 (30·8%)  18 (69·2%) |
| Spinal surgery  No  Yes  Unknown | 12 (40·0%)  7 (23·3%)  11 (36·7%) | 12 (40·0%)  7 (23·3%)  11 (36·7%) | 14 (51·9%)  6 (22·2%)  7 (25·9%) | 11 (42·3%)  7 (26·9%)  8 (30·8%) |
| Baseline HFMSE  ≥35  <35  >60  <5  Median baseline HFMSE  Mean baseline HFMSE | 9 (30·0%)  21 (70·0%)  2 (6·7%)  10 (33·3%)  12·5 (1·0-49·5)  22·8 (23·0) | 10 (33·3%)  20 (66·7%)  2 (6·7%)  12 (40·0%)  10·0 (0·8-39·3)  21·1 (23·2) | 10 (37·0%)  17 (63·0%)  1 (3·7%)  10 (37·0%)  12·0 (1·0-49·0)  23·2 (22·7) | 8 (30·8%)  18 (69·2%)  0 (0·0%)  11 (42·3%)  8·0 (0·8-35·3)  18·7 (20·7) |
| Patients with clinically meaningful improvement in HFMSE | 5 (16·7%) | 7 (23·3%) | 8 (29·6%) | 8 (30·8%) |

Data are n (%), mean (SD) or median (IQR). HFMSE=Hammersmith Functional Motor Scale Expanded. RULM=Revised Upper Limb Module.

**Figure** Individual trajectories of absolute (non-normalized) values of **(A)** HFMSE, **(B)** RULM, and **(C)** ALSFRS-R. ALSFRS-R=Amyotrophic Lateral Sclerosis Functional Rating Scale Revised. HFMSE=Hammersmith Functional Motor Scale Expanded. RULM=Revised Upper Limb Module.

**Table** Characteristics of patients included in the RULM analysis.

|  | **Included in T1 analysis (n=36)** | **Included in T2 analysis (n=40)** | **Included in T3 analysis (n=35)** | **Included in T4 analysis (n=34)** |
| --- | --- | --- | --- | --- |
| Female | 20 (55·6%) | 19 (47·5%) | 19 (54·3%) | 16 (47·1%) |
| Age at first therapy, years | 36·5 (28·8-43·7) | 35·6 (29·7-43·7) | 35·7 (29·7-41·3) | 35·0 (29·3-42·1) |
| Treatment time until last examination, months | 22·5 (13·3-24·0) | 23·0 (15·3-24·8) | 23·0 (18·0-24·0) | 23·0 (22·0-25·3) |
| Disease duration before treatment, years  Patients with available  information | 28·5 (22·3-36·3)  27 (75·0%) | 30·1 (24·7-38·6)  33 (82·5%) | 30·3 (24·2-37·2)  25 (71·4%) | 29·0 (23·9-35·4)  27 (79·4%) |
| Time from treatment start to analysis, months | 3·5 (3·0-4·0) | 9·0 (8·0-11·0) | 15·0 (14·0-16·0) | 24·0 (21·3-26·0) |
| Spinal muscular atrophy type  Type I  Type II  Type III  Type IV  Unknown | 1 (2·8%)  15 (41·7%)  15 (41·7%)  4 (11·1%)  1 (2·8%) | 2 (5·0%)  18 (45·0%)  17 (42·5%)  2 (5·0%)  1 (2·5%) | 2 (5·7%)  13 (37·1%)  18 (51·4%)  2 (5·7%)  0 (0·0%) | 2 (5·9%)  14 (41·2%)  16 (47·1%)  2 (5·9%)  0 (0·0%) |
| *SMN2* copies  1  2  3  4  Unknown | 1 (2·8%)  2 (5·6%)  21 (58·3%)  10 (27·8%)  2 (5·6%) | 1 (2·5%)  3 (7·5%)  23 (57·5%)  13 (32·5%)  0 (0·0%) | 1 (2·9%)  2 (5·7%)  20 (57·1%)  9 (25·7%)  3 (8·6%) | 0 (0·0%)  2 (5·9%)  20 (58·8%)  10 (29·4%)  2 (5·9%) |
| Ambulatory status  Ambulatory  Non-ambulatory | 6 (16·7%)  30 (83·3%) | 6 (15·0%)  34 (85·0%) | 7 (20·0%)  28 (80·0%) | 6 (17·6%)  28 (82·4%) |
| Spinal surgery  No  Yes  Unknown | 13 (36·1%)  12 (33·3%)  11 (30·6%) | 14 (35·0%)  16 (40·0%)  10 (25·0%) | 18 (51·4%)  10 (28·6%)  7 (20·0%) | 17 (50·0%)  9 (26·5%)  8 (23·5%) |
| Baseline HFMSE  ≥35  <35  Unknown | 5 (13·9%)  22 (61·1%)  9 (25·0%) | 6 (15·0%)  21 (52·5%)  13 (32·5%) | 7 (20·0%)  19 (54·3%)  9 (25·7%) | 6 (17·6%)  21 (61·8%)  7 (20·6%) |
| Baseline RULM  >35  <10  Median baseline RULM  Mean baseline RULM | 6 (16·7%)  12 (33·3%)  17·0 (4·3-25·8)  16·9 (12·7) | 5 (12·5%)  19 (47·5%)  12·5 (2·5-23·5)  13·9 (12·9) | 5 (14·3%)  12 (34·3%)  17·0 (3·0-27·0)  16·6 (13·2) | 5 (14·7%)  12 (35·3%)  16·0 (2·5-26·3)  16·3 (13·1) |
| Patients with clinically meaningful improvement in RULM | 12 (33·3%) | 14 (35·0%) | 17 (48·6%) | 18 (52·9%) |

Data are n (%), mean (SD) or median (IQR). HFMSE=Hammersmith Functional Motor Scale Expanded. RULM=Revised Upper Limb Module.

**Table** Characteristics of patients included in the ALSFRS-R analysis.

|  | **Included in T1 analysis (n=27)** | **Included in T2 analysis (n=32)** | **Included in T3 analysis (n=29)** | **Included in T4 analysis (n=28)** |
| --- | --- | --- | --- | --- |
| Female | 14 (51·9%) | 15 (46·9%) | 16 (55·2%) | 14 (50·0%) |
| Age at first therapy, years | 31·8 (26·7-42·8) | 33·8 (29·5-42·6) | 32·6 (29·3-40·8) | 31·9 (28·9-40·8) |
| Treatment time until last examination, months | 23·0 (15·0-25·0) | 23·0 (18·0-25·0) | 23·0 (18·5-25·0) | 24·0 (22·0-25·8) |
| Disease duration before treatment, years  Patients with available  information | 27·1 (22·7-26·1)  24 (88·9%) | 29·7 (24·3-35·4)  28 (87·5%) | 29·3 (23·9-34·8)  22 (75·9%) | 28·8 (24·0-34·4)  24 (85·7%) |
| Time from treatment start to analysis, months | 4·0 (3·0-4·0) | 9·0 (8·0-11·0) | 14 (13·5-16·0) | 24·0 (21·0-26·0) |
| Spinal muscular atrophy type  Type I  Type II  Type III  Type IV | 1 (3·7%)  11 (40·7%)  12 (44·4%)  3 (11·1%) | 2 (6·3%)  14 (43·8%)  14 (43·8%)  2 (6·3%) | 2 (6·9%)  11 (37·9%)  14 (48·3%)  2 (6·9%) | 2 (7·1%)  12 (42·9%)  12 (42·9%)  2 (7·1%) |
| *SMN2* copies  1  2  3  4  Unknown | 1 (3·7%)  1 (3·7%)  16 (59·3%)  8 (29·6%)  1 (3·7%) | 1 (3·1%)  2 (6·3%)  19 (59·4%)  10 (31·3%)  0 (0·0%) | 1 (3·4%)  2 (6·9%)  16 (55·2%)  8 (27·6%)  2 (6·9%) | 0 (0·0%)  2 (7·1%)  17 (60·7%)  8 (28·6%)  1 (3·6%) |
| Ambulatory status  Ambulatory  Non-ambulatory | 5 (18·5%)  22 (81·5%) | 6 (18·8%)  26 (81·3%) | 8 (27·6%)  21 (72·4%) | 7 (25·0%)  21 (75·0%) |
| Spinal surgery  No  Yes  Unknown | 12 (44·4%)  10 (37·0%)  5 (18·5%) | 15 (46·9%)  11 (34·4%)  6 (18·7%) | 16 (55·2%)  9 (31·0%)  4 (13·8%) | 15 (53·6%)  9 (32·1%)  4 (14·3%) |
| Baseline HFMSE  ≥35  <35  Unknown | 4 (14·8)  18 (66·7)  5 (18·5) | 6 (18·7%)  18 (56·3%)  8 (25·0%) | 8 (27·6%)  16 (55·2%)  5 (17·2%) | 7 (25·0%)  17 (60·7%)  4 (14·3%) |
| Median baseline ALSFRS-R  Mean baseline ALSFRS-R | 30·0 (23·0-39·0)  30·9 (9·2) | 27·5 (22·3-37·0)  29·5 (9·7) | 32·0 (23·0-41·0)  31·7 (10·0) | 30·5 (21·5-39·8)  31·0 (10·4) |

Data are n (%), mean (SD) or median (IQR). HFMSE=Hammersmith Functional Motor Scale Expanded. ALSFRS-R=Amyotrophic Lateral Sclerosis Functional Rating Scale Revised.

**Figure (A-D)** Changes in ALSFRS-R from baseline at the predefined timepoints, with bars representing individual patients. Reported p-values correspond to the Wilcoxon signed rank test. **(E)** Mean pre-post changes (with standard error) in ALSFRS-R at each predefined timepoint. **(F)** Individual ALSFRS-R trajectories (red) after treatment initiation, with baseline scores normalised to 0. The LOESS smoothing line (black) estimates average ALSFRS-R change over time. ALSFRS-R=Amyotrophic Lateral Sclerosis Functional Rating Scale Revised. CI=Confidence Interval. LOESS=Locally Estimated Scatterplot Smoothing.

**Table** Characteristics of patients included in the 6MWT analysis.

|  | **Included in T1 analysis (n=9)** | **Included in T2 analysis (n=10)** | **Included in T3 analysis (n=10)** | **Included in T4 analysis (n=7)** |
| --- | --- | --- | --- | --- |
| Female | 7 (77·8%) | 8 (80·0%) | 7 (70·0%) | 5 (71·4%) |
| Age at first therapy, years | 39·0 (28·1-53·8) | 37·3 (28·8-47·6) | 32·3 (28·4-39·6) | 32·6 (29·5-41·3) |
| Treatment time until last examination, months | 23·0 (8·5-24·5) | 22·5 (15·3-24·3) | 23·5 (17·8-25·3) | 24·0 (22·0-25·0) |
| Disease duration before treatment, years  Patients with available  information | 24·6 (20·7-32·8)  7 (77·8%) | 28·8 (21·4-33·7)  8 (80·0%) | 26·5 (21·4-32·8)  8 (80·0%) | 26·5 (22·9-30·3)  6 (85·7%) |
| Time from treatment start to analysis, months | 3·0 (3·0-4·5) | 9·5 (7·0-11·0) | 15·5 (13·8-16·3) | 24·0 (23·0-25·0) |
| Spinal muscular atrophy type  Type I  Type II  Type III  Type IV | 0 (0·0%)  0 (0·0%)  7 (77·8%)  2 (22·2%) | 0 (0·0%)  0 (0·0%)  9 (90·0%)  1 (10·0%) | 0 (0·0%)  0 (0·0%)  9 (90·0%)  1 (10·0%) | 0 (0·0%)  0 (0·0%)  6 (85·7%)  1 (14·3%) |
| *SMN2* copies  1  2  3  4 | 0 (0·0%)  1 (11·1%)  1 (11·1%)  7 (77·8%) | 0 (0·0%)  1 (10·0) %  3 (30·0%)  6 (60·0%) | 0 (0·0%)  1 (10·0%)  3 (30·0%)  6 (60·0%) | 0 (0·0%)  0 (0·0%)  3 (42·9%)  4 (57·1%) |
| Spinal surgery  No  Yes  Unknown | 5 (55·6%)  0 (0·0%)  4 (44·4%) | 6 (60·0%)  0 (0·0%)  4 (40·0%) | 7 (70·0%)  0 (0·0%)  3 (30·0%) | 4 (57·1%)  0 (0·0%)  3 (42·9%) |
| Median baseline 6MWT, meters  Mean baseline 6MWT, meters | 490·0 (395·0-538·0)  477·8 (137·8) | 417·5 (146·5-515·3)  374·5 (191·7) | 412·5 (146·5-515·3)  369·5 (189·2) | 390·0 (136·0-576·0)  371·4 (204·9) |

Data are n (%), mean (SD) or median (IQR). 6MWT=6-Minute Walk Test.

**Figure** Bland-Altman blot of HFMSE T0 vs. T4.HFMSE=Hammersmith Functional Motor Scale Expanded. σ= Standard Deviation

**Figure** HFMSE trajectories of subgroups after treatment initiation based on (**A)** SMA type, (**B)** HFMSE at baseline, **(C)** ambulatory status, and (**D)** history of spinal surgery. Faint lines represent individual patients, bold lines represent LOESS curves. HFMSE=Hammersmith Functional Motor Scale Expanded. LOESS=Locally Estimated Scatterplot Smoothing. SMA=Spinal Muscular Atrophy.

**Table** Adverse events.

| **Adverse event** | **Number of patients (%)** |
| --- | --- |
| Total number of patients with adverse reactions  Dry or sensitive skin  Gastrointestinal  Elevated amylase and lipase (<2x the upper limit of normal)  Nausea  Headache  Concentrated urine  Bone fracture  Acute cardiac event  Infection (dental) | 8 (14·0%)  3 (37·5%)  2 (25·0%)  1 (12·5%)  1 (12·5%)  1 (12·5%)  1 (12·5%)  1 (12·5%)  1 (12·5%)  1 (12·5%) |

**References**

1. Sitas B, Hancevic M, Bilic K, et al. Risdiplam Real World Data - Looking Beyond Motor Neurons and Motor Function Measures. *J Neuromuscul Dis* 2024; 11: 75-84. DOI: 10.3233/JND-230197.

2. Severa G, Alfaro MDC, Alimi Ichola C, et al. Risdiplam: therapeutic effects and tolerability in a small cohort of 6 adult type 2 and type 3 SMA patients. *Orphanet J Rare Dis* 2024; 19: 430. 20241120. DOI: 10.1186/s13023-024-03442-0.

3. McCluskey G, Lamb S, Mason S, et al. Risdiplam for the treatment of adults with spinal muscular atrophy: Experience of the Northern Ireland neuromuscular service. *Muscle Nerve* 2023; 67: 157-161. 20221205. DOI: 10.1002/mus.27755.

4. Kessler T, Sam G, Wick W, et al. Evaluation of risdiplam efficacy in 5q spinal muscular atrophy: A systematic comparison of electrophysiologic with clinical outcome measures. *Eur J Neurol* 2024; 31: e16099. 20231012. DOI: 10.1111/ene.16099.

5. Nungo Garzon NC, Pitarch Castellano I, Sevilla T, et al. Risdiplam in non-sitter patients aged 16 years and older with 5q spinal muscular atrophy. *Muscle Nerve* 2023; 67: 407-411. 20230317. DOI: 10.1002/mus.27804.

6. Gavriilaki M, Moschou M, Pagiantza M, et al. Risdiplam in Adult Patients With 5q Spinal Muscular Atrophy: A Single-Center Longitudinal Study. *Muscle Nerve* 2024 20241226. DOI: 10.1002/mus.28327.

7. Brakemeier S, Lipka J, Schlag M, et al. Risdiplam improves subjective swallowing quality in non-ambulatory adult patients with 5q-spinal muscular atrophy despite advanced motor impairment. *J Neurol* 2024; 271: 2649-2657. 20240215. DOI: 10.1007/s00415-024-12203-9.

8. Bjelica B, Wohnrade C, Cespedes I, et al. Risdiplam therapy in adults with 5q-SMA: observational study on motor function and treatment satisfaction. *BMC Neurol* 2024; 24: 67. 20240217. DOI: 10.1186/s12883-024-03562-x.

9. Iterbeke L and Claeys KG. Two-year Risdiplam treatment in adults with spinal muscular atrophy: improvements in motor and respiratory function, quality of life and fatigue. *Neuromuscul Disord* 2025; 52: 105397. 20250528. DOI: 10.1016/j.nmd.2025.105397.
